# Blood circulating exosomes carrying microRNA-423-5p regulates cell progression in prostate cancer via targeting FRMD3

**DOI:** 10.7150/jca.71706

**Published:** 2022-07-18

**Authors:** Yongbao Wei, Zhensheng Chen, Ruochen Zhang, Bangkui Wu, Le Lin, Qingguo Zhu, Liefu Ye, Tao Li, Feng Li

**Affiliations:** 1 Shengli Clinical Medical College of Fujian Medical University, Fuzhou, China; 2 Department of Urology, Fujian Provincial Hospital, Fuzhou, China; 3 Department of Urology, Fuding Hospital Affiliated to Fujian University of Traditional Chinese Medicine, Fuding, Fujian Province, China; 4 Shanghai Engineering Research Center of Pharmaceutical Translation, Shanghai, China; 5 Department of Pathology, Fujian Provincial Hospital, Fuzhou, China

**Keywords:** microRNA-423-5p, FRMD3, prostate cancer, exosome, proliferation

## Abstract

This study explored the role of circulating exosomal microRNA-423-5p in the progression of PCa and its molecular mechanism. First, based on the microarray analysis, microRNA-423-5p was at a high expression level in PCa peripheral blood samples. It was demonstrated that microRNA-423-5p expression in serum exosomes of PCa patients was notably higher than that in healthy people as revealed by qRT-PCR. Further studies indicated that overexpressing microRNA-423-5p promoted cell progression of PCa. Microarray analysis and luciferase gene reporter assay illustrated that FRMD3 was targeted by microRNA-423-5p, and its expression was down-regulated by microRNA-423-5p. While FEMD3 knockdown would reverse the repressive effect of silencing microRNA-423-5p on PCa cell functions. In addition, it was exhibited that exosomes carrying microRNA-423-5p could internalize into PCa cells by labeling and tracing exosomes. Cell function assays and animal experiments manifested those exosomes carrying microRNA-423-5p could enhance PCa cell proliferation, migration, and invasion *in vivo*. In conclusion, this study indicated that blood circulating exosomal microRNA-423-5p played important roles in PCa cell functions, and illustrated the molecular mechanism of microRNA-423-5p as an oncogene in PCa.

## Introduction

Prostate cancer (PCa) is a frequent aggressive tumor in men 1. It is estimated that there will be more than 1.7 million PCa cases with 499,000 new deaths by 2030 because of the rapid growth and worse aging of the global population 2. Most patients with PCa are in the advanced stage at the time of diagnosis because of unobvious symptoms 3. Moreover, bone metastases account for a high proportion of PCa patients at initial diagnosis in China, accounting for 13.3%-26% of all confirmed cases, which seriously threats the life safety of patients 4,5. Increasing evidence suggested that microRNAs in serum exosomes can serve as an alternative non-invasion biopsy method except for prostate-specific antigen (PSA) 6. Thus, starting from the serum exosomes, it is of great meaning to look for abnormally expressed molecules for an in-depth understanding of the mechanism of PCa development and progression.

It was manifested that the tumor microenvironment (TME) is involved in the malignant transformation of cancers including PCa 7-9. Exosomes, one of the main subtypes of Extracellular Vehicles (EVs), are widely found in the urine, blood, and other body fluids 10,11. Exosomes are 30-100 nm EVs enclosed by a lipid bilayer, which contains many kinds of active molecules, like proteins, mRNAs, and non-coding RNA 6,12,13. Current studies indicated that EVs, especially exosomes, play crucial roles in the process of intercellular communication. The main function of exosomes is to act as an intercellular transport system to transfer the contents of cancer cells, especially microRNAs 13,14. It was reported that much plasma exosome-carried RNA can be biomarkers for diagnosis, treatment target, and prognosis biomarker of cancer patients 15. Divya Bhagirath et al. 16 proved that microRNA-1246 is a PCa biomarker with diagnostic potential and can predict the invasiveness of disease. Plasma exosomes carrying microRNA-125a-5p and microRNA-141-5p serve as non-invasive biomarkers for PCa 17. Moreover, compared with benign prostate hyperplasia patients or healthy volunteers, serum exosomal microRNA-141 of PCa patients is upregulated, which may be an underlying biomarker for diagnosis of metastatic PCa 6. Apart from their diagnostic value, microRNAs in serum exosomes contributes to detecting patients' therapeutic response 18. In conclusion, these studies all exhibited the great significance of serum exosomal microRNAs for the treatment, diagnosis, and prognosis of PCa patients.

It was reported that microRNA-423-5p is one of the most common tumor-related microRNAs, and it is abnormally expressed in a series of cancers, such as osteosarcoma, colon cancer, breast cancer, ovarian cancer, etc. 19-22. MicroRNA-423-5p enhances cell proliferation and inhibits cell apoptosis in PCa by modulating GRIM-19 23. Nevertheless, whether microRNA-423-5p is enriched in circulating exosomes of PCa patients and its function in PCa remain unclear. Furthermore, whether microRNA-423-5p can target other downstream genes in addition to GRIM-19 in cancer needs to be investigated. We thereby explored the expression level of blood circulating exosomal microRNA-423-5p of PCa patients and the functions of microRNA-423-5p in cancer progression, to illustrate its potential mechanism.

## Materials and methods

### Microarray analysis

GSE61741 (Normal:94, PCa:65) peripheral blood microRNA microarray data were offered by Gene Expression Omnibus (GEO) database. R package 'limma' was used for differential expression analysis (|logFC|>1.0, padj<0.05), combined with literature citation to determine the target microRNA. 'EVmiRNA' was utilized to verify the expression level of target exosomal microRNA in the blood of PCa patients.

The expression data of mature microRNAs (normal: 52, tumor:499) and mRNAs (normal: 52, tumor:499) were downloaded from The Cancer Genome Atlas (TCGA) database. Expression analyses were performed by the 'edgeR' package, thus obtaining differential microRNAs (|logFC|>1.0, padj<0.05) and mRNAs (|logFC|>1.0, padj<0.05). StarBase, mirDIP, and miRDB databases were utilized to predict the downstream targets of the researched microRNA, and then the intersection with differential mRNA was taken to obtain the target gene with binding sites with the target microRNA.

### Samples of patients

This study got the approval from the Ethics Committee of our hospital; and written informed consent from the participants. Peripheral blood samples of the control group were volunteers without malignant tumors (n=20) and PCa patients (n=56) in our hospital between 2019 and 2021 were included. 56 PCa patients received no treatment before surgery, whose median age was 77.5 years old. The patients were only included in this study when they met all of the following inclusion criteria: (a) they were PCa- related treatment naïve; (b) the diagnosis of PCa was confirmed by pathological examination; (c) their detailed clinical and pathological information was available and informed consents were obtained. If they had other hematological malignancies or solid malignant tumors were excluded. The control group was male patients with other diseases hospitalized during the same period, with a median age of 72.5 years old. These patients with the following non-neoplastic diseases were eligible: urinary stones, ureteropelvic junction obstruction or benign prostatic hyperplasia, etc.; they were excluded malignant tumors through medical history, and physical and laboratory examinations (Supplementary Table 1). The collected blood was centrifuged at 3×10^3^ g for 10 min, then the supernatant (serum) was transferred to RNase-free tubes and maintained at -80 °C for further use.

### Exosomes extraction

After being extracted from human serum with an ExoQuick exosome precipitation solution kit (EXOQ5A-1; System Biosciences, USA), exosomes were kept at -80 °C or treated immediately to extract microRNA-423-5p.

### Transmission electron microscope (TEM)

Briefly, exosome suspension was supplemented into paraformaldehyde and loaded into a carbon-supported grid of TEM (Alliance Biosystems, Inc, Japan). Next, glutaric dialdehyde was utilized to fix samples, uranyl acetate was used for staining and phenolic epoxy resin was adopted to embed samples. After polymerization, the exosomes were photographed using the Hitachi H-7650 TEM (Hitachi, Ltd, Japan).

### Cell culture and transfection

PCa cell lines LNCaP (ATCC® CRL-1740), PC-3 (ATCC® CRL-1435), DU145 (ATCC® HTB-81), human epidermal cell line RWPE‐1 (ATCC® CRL-11609) were all bought from American Type Culture Collection (ATCC; USA). PC-3 was cultured in an F-12 medium. LNCaP and RWPE-1 were cultivated in Roswell Park Memorial Institute-1640 (RPMI-1640) medium. DU145 was cultured in Eagle's Minimum Essential medium. 10% fetal bovine serum (FBS), streptomycin (0.1 mg/mL), and penicillin (100 U/mL) were appropriately supplemented to the mediums. All cells were incubated under routine conditions in the humidified incubator. Cells were then transfected with specific nucleotides or plasmids using Lipofectamine 2000 (Invitrogen, USA).

### Lentivirus expression vector and cell transfection

Overexpression vectors LV-microRNA-423-5p mimic (LV2-miR-423-5p) was established by utilizing Lentivirus expression vector pLVX-IRES-neo (Clontech Inc., USA). While pLVX-IRES-neo vector (LV-NC) was applied as the negative control. After being treated with 5µg/mL puromycin for 5 days, the transfected cells were screened to obtain the stably transfected cell lines.

### qRT-PCR

Exosomal microRNA in serum was isolated using miRNeasy Serum/Plasma Kit (Qiagen, Germany). The extracted microRNA was reversely transcribed using the miScript II RT kit (Qiagen, Germany) and its expression was detected using the miScript SYBR Green PCR kit (Qiagen, Germany). Total RNA in PCa cells was separated using Trizol reagent (Invitrogen). SYBR-Green PCR Master Mix kit was introduced for qRT-PCR. And qRT-PCR was performed on ABI7500 quantitative PCR apparatus (Thermo Fisher Scientific, USA). U6 and GAPDH were internal references. Primer sets = displayed in Table 1 were designed by Shanghai GenePharma Co. Ltd. (China).

### Western blot

Exosomes and cells were lysed using radioimmunoprecipitation assay lysis buffer containing protein inhibitors. An equivalent quantity of proteins (15 μg) was electrophorized by sodium dodecyl sulfate-polyacrylamide gel electrophoresis; and then transferred to a polyvinylidene fluoride (Bio-Rad Laboratories, Inc) membrane. After being blocked in 5% (w/v) skimmed milk, the membrane was incubated with primary antibodies overnight at 4 °C. The primary antibodies were: FRMD3 (ab166071, 1:1000), CD-9 (ab92726, 1:2000), CD-63 (ab134045, 1:10000), TSG101 (ab125011, 1:1000), E-cadherin (ab40772, 1:10000), N-cadherin (ab76011, 1:5000), Vimentin (ab92547, 1:1000) and GAPDH (ab9485, 1:2500). Subsequently, the membrane was reacted with horseradish peroxidase-labeled goat anti-rabbit secondary antibody IgG H&L (ab6721, 1:2000) for 1h. All antibodies were bought from Abcam Company (UK). All bands were measured by electrochemiluminescence (ECL) western blot kit (Amersham Biosciences, Little Chalfont, UK) and processed by Image Lab 6.0.1 Software (Bio-Rad Laboratories, Inc.).

### Cell proliferation detection

Cell counting kit-8 (CCK-8; Beyotime, China) was used to detect cell viability. 5×10^3^ PCa cells were planted into 96-well plates. 10 μl CCK-8 reagent was respectively supplemented to each well for 24, 48, and 72 h, then the cells were stored at 37 °C for 4 h. Finally, the absorbance was measured at 450 nm by a microplate reader (Tecan Austria, GmbH, Grodig, Austria) to analyze cell viability.

For cell colony formation assay, cells (500 cells per well) were plated into 12-well plates and stored at 37 °C with 5% CO_2_ for 2 weeks. Cells were fixed with methanol and stained with 0.1% crystal violet for 20 min. The colonies were counted with ImagePro Plus 6.0 software (Media Cybernetics, USA).

### Transwell assay

For the invasion detection, 50 μl Matrigel U5Gl (BD Biosciences, Franklin Lakes, NJ) was pre-coated in the upper chamber. 2×10^4^ cells suspended in 100 μl serum-free medium were put into the upper layer of the Transwell chamber (BD Biosciences). 500 μl medium with 10% FBS was added into the lower chamber. After 48-h cell incubation, the non-invading cells were removed, and invading cells were treated with methanol and 0.1% crystal violet. Micrographs were obtained from an inverted microscope (XDS-800D, Shanghai Caikon Optical Instrument Co., Ltd., China), and invading cells in random regions were counted. The cell migration assay was similar to the above assay except that Matrigel was not required in the upper Transwell chamber.

### Dual-luciferase assay

3'UTR of FRMD3 with microRNA-423-5p binding sites (FRMD3-wild type (WT)) and 3'UTR of mutant FRMD3 (FRMD3-WT) were cloned into pmiR-RB-Report (Promega, China). Next, the constructed vectors and microRNA-423-5p mimic or mimic NC were transfected together into cells using lipofectaminetm3000 (Invitrogen). After being co-transfected for 48h, the dual-luciferase assay kit (Promega) was utilized to test luciferase activity.

### Cellular uptake of exosome assay

Exosomes (20 µg) were marked by Carboxyfluorescein succinimidyl ester (CFSE) and diluted at 1:1000. After incubation for 15 min, the exosomes were rinsed with phosphate-buffered saline (PBS), then centrifugated at 1×10^5^ g for 70 min, and finally collected. The CFSE-labeled exosomes were incubated with PCa cells at 37 °C for 6, 18, and 36 h to observe cell uptake with a confocal fluorescence microscope.

### Tumor mouse models

24 BALB/c athymic male nude mice aged from 6 to 8 weeks were purchased from Shanghai Lab. Animal Research Center, Chinese Academy of Sciences (China), and raised under specific pathogen-free conditions. Nude mice were randomly grouped into LV-NC, LV-miR-423-5p, PBS, and exosome, with 6 mice in each group, and tumor growth was observed. Cells in each group were suspended with 50% Matrigel (BD Biosciences, USA). 0.2 mL cells (1×10^6^ cells /mL) were subcutaneously injected into the flank of mice. After being injected for 8 days, tumor size was measured weekly with vernier calipers, and tumor volume (mm^3^) was calculated according to formula 1/2 (length×width^2^). After subcutaneous injection for 4 weeks, all nude mice were sacrificed, and their tumors were taken and weighed. A portion of the tumor was detected by qRT-PCR and another portion was sectioned for immunohistochemical observation.

### Immunohistochemistry

Paraffin-embedded tissue sections were fixed with formalin, dewaxed in dimethylbenzene, rehydrated with graded ethanol, and then boiled in citric acid buffer (10 mM, pH 6.0) for 30 min to extract antigens. Then the tissue was blocked and sectioned with 5% albumin from bovine serum (Boster Bioengineering, China). Subsequently, the diluted primary antibody Ki-67 (ab21700, 1:150, Abcam, UK) was added into the sections which were then incubated overnight at 4 °C, and then secondary antibody IgG H&L (HRP) (ab6721, 1:2000) was added to incubate the sections for 1h at 37 °C. The protein was stained with DAB and the nucleus was re-stained with hematoxylin. Finally, an optical microscope was introduced to observe staining (Olympus).

### Data analysis

SPSS 21.0 software (IBM Corp. USA) was utilized to analyze data. All experiments were conducted for at least 3 replications. The results were presented in the form of mean ± standard deviation. T-test or one-way ANOVA analysis of variance was utilized to compare differences between or among groups. *P*<0.05 indicated statistically significant.

## Results

### MicroRNA-423-5p is up-regulated in serum exosomes in patients with PCa

**Figure 1:**
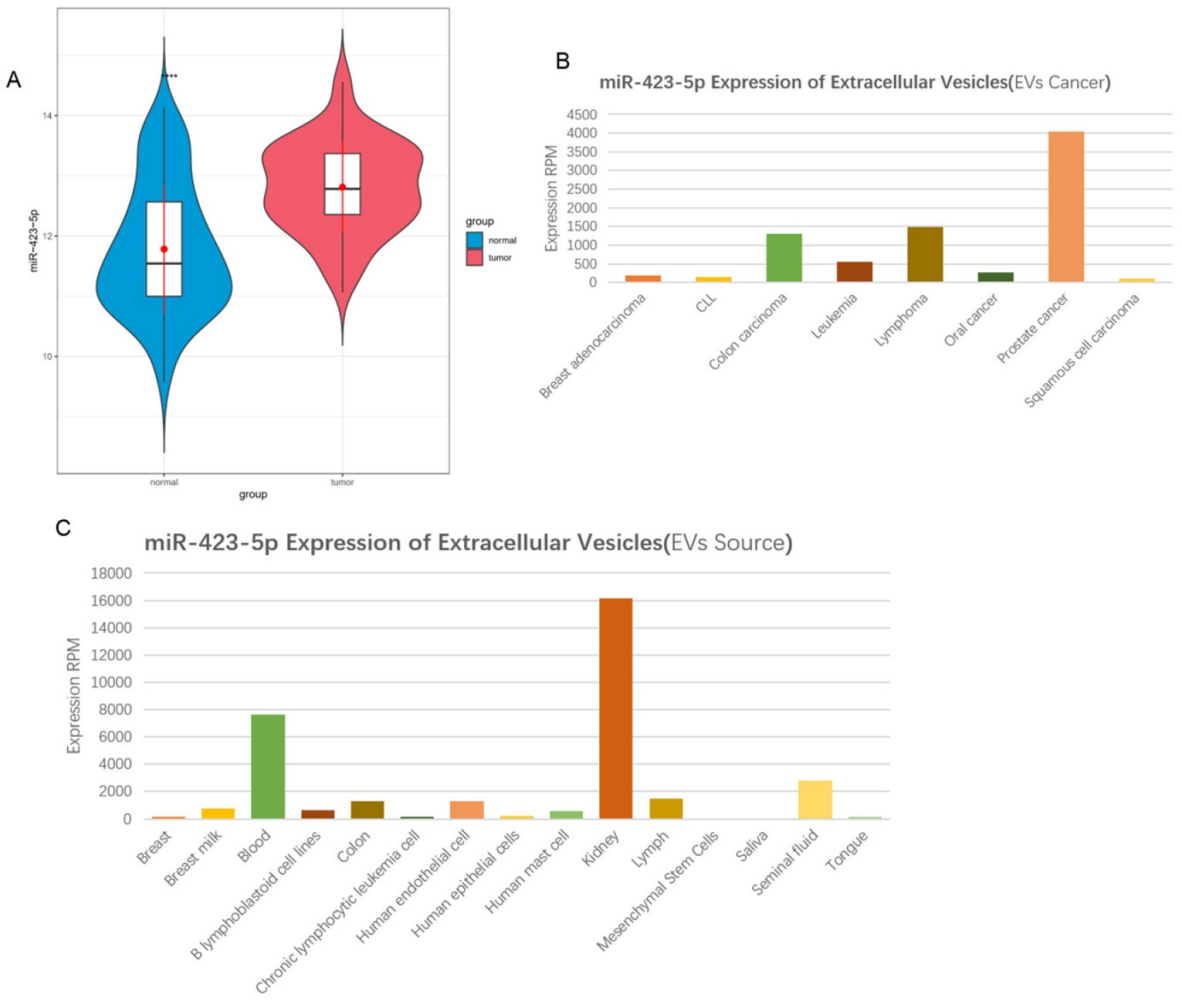
**MicroRNA-423-5p is up-regulated in serum exosomes in patients with PCa**. A: MicroRNA-423-5p expression in GSE61741 dataset, with green boxplot representing normal peripheral blood samples and red boxplot representing tumor peripheral blood samples; B-C: MicroRNA-423-5p expression in 'EVmiRNA' database.

According to the information in the GEO database, it was shown that microRNA-423-5p expression was high in PCa peripheral blood samples (Figure 1A). Exosomal microRNA-423-5p was highly expressed in PCa patients based on the 'EVmiRNA' database (Figure 1B) and largely existed in the blood (Figure 1C). A previous study exhibited that the high expression of blood exosomal microRNA-423-5p in patients with gastric cancer promotes cancer growth, and the exosomal microRNA-423-5p can act as a novel biomarker for this cancer 24. Based on the above findings, we speculated that microRNA-423-5p in the blood of PCa patients may originate from blood exosomes and can regulate the malignant progression of PCa. Thus, blood exosomal microRNA-423-5p was selected as the subject for further study.

### MicroRNA-423-5p is at a high expression level in blood exosomes in PCa patients

**Figure 2:**
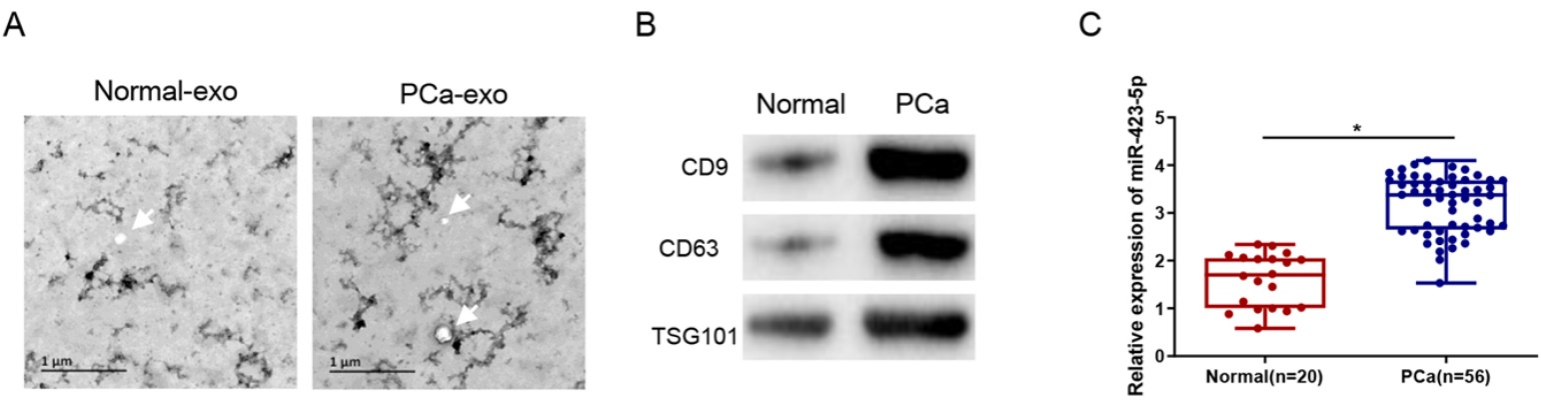
** MicroRNA-423-5p is highly expressed in blood exosomes in PCa patients.** A: Exosomes extracted from blood samples of healthy people and PCa patients were observed by TEM. Bar=200nm; B: The expressions of labeled proteins (CD9, CD63 and TGS101) in blood exosomes; C: The expression of microRNA-423-5p in blood exosomes of healthy people and PCa patients. (*p<0.05)

Exosomes were isolated from the blood of clinical samples, and the physical characteristics and molecular markers of the isolated exosomes were characterized. The exosomes were observed by TEM as round or oval vesicles (diameters: 30 to 100 nm), and the membrane structure was intact (Figure 2A). Western blot results displayed that the protein level of CD9, CD63, and TSG101 was prominently elevated in PCa patients (Figure 2B). These results proved the successful isolation of exosomes from human blood samples. Additionally, the qRT-PCR result exhibited that the exosomal microRNA-423-5p level was notably higher in the blood of PCa patients than that in the control group (normal) (Figure 2C).

### MicroRNA-423-5p-enriched blood exosomes facilitate the malignant progression of PCa

**Figure 3:**
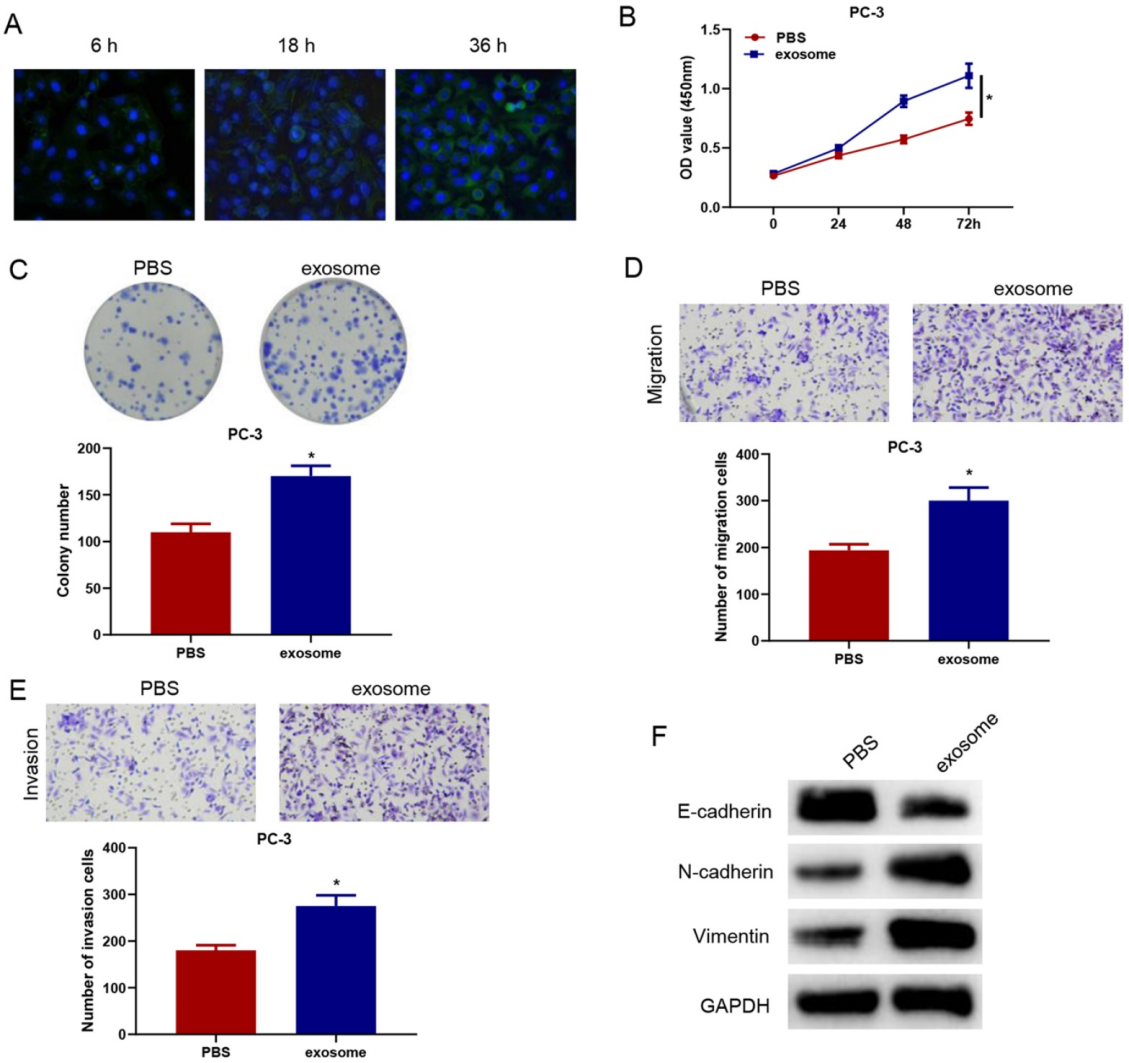
** MicroRNA-423-5p in blood exosomes of PCa patients promotes cancer progression.** A: Exosome uptake in PC-3 cells was observed by a confocal fluorescence microscope (scale bar= 25 µM); B: Cell activity of each group; C: Colony formation in treatment groups; D-E: Cell migration and invasion in different groups; F: The expression of EMT-related proteins of each group. (* *p*<0.05)

To verify whether serum exosomal microRNA-423-5p could enter and affect the activity of PCa cells, we labeled blood exosomes of PCa patients with CFSE and then incubated the labeled exosomes with PC-3 cells. The internalization of exosomes in PC-3 cells was observed by a confocal fluorescence microscope. CFSE-marked exosomes were found to be localized in the cytoplasm. After being incubated for 36h, almost all PC-3 cells turned green, suggesting that PC-3 cells significantly ingested CFSE-labeled exosomes (Figure 3A).

PC-3 cells were co-incubated with exosomes or PBS for 48 h to observe the changes in cell biological functions. Compared with the PBS+PC-3 group, cells in the exosome+PC-3 (exo+PC-3) group had stronger cell viability and colony formation ability (Figure 3B-C). Transwell assay confirmed that, compared with the PBS+PC-3 group, the number of migrating and invading cells in the exo+PC-3 group increased significantly (Figure 3D-E). Besides, compared to the control group, E-cadherin expression was reduced, while N-cadherin and Vimentin expression was elevated in the exo+PC-3 group (Figure 3F). These data exhibited that exosomes could enter PC-3 cells, and accelerate the progression of cancer cells.

### MicroRNA-423-5p accelerates proliferation, migration, and invasion of PCa

**Figure 4:**
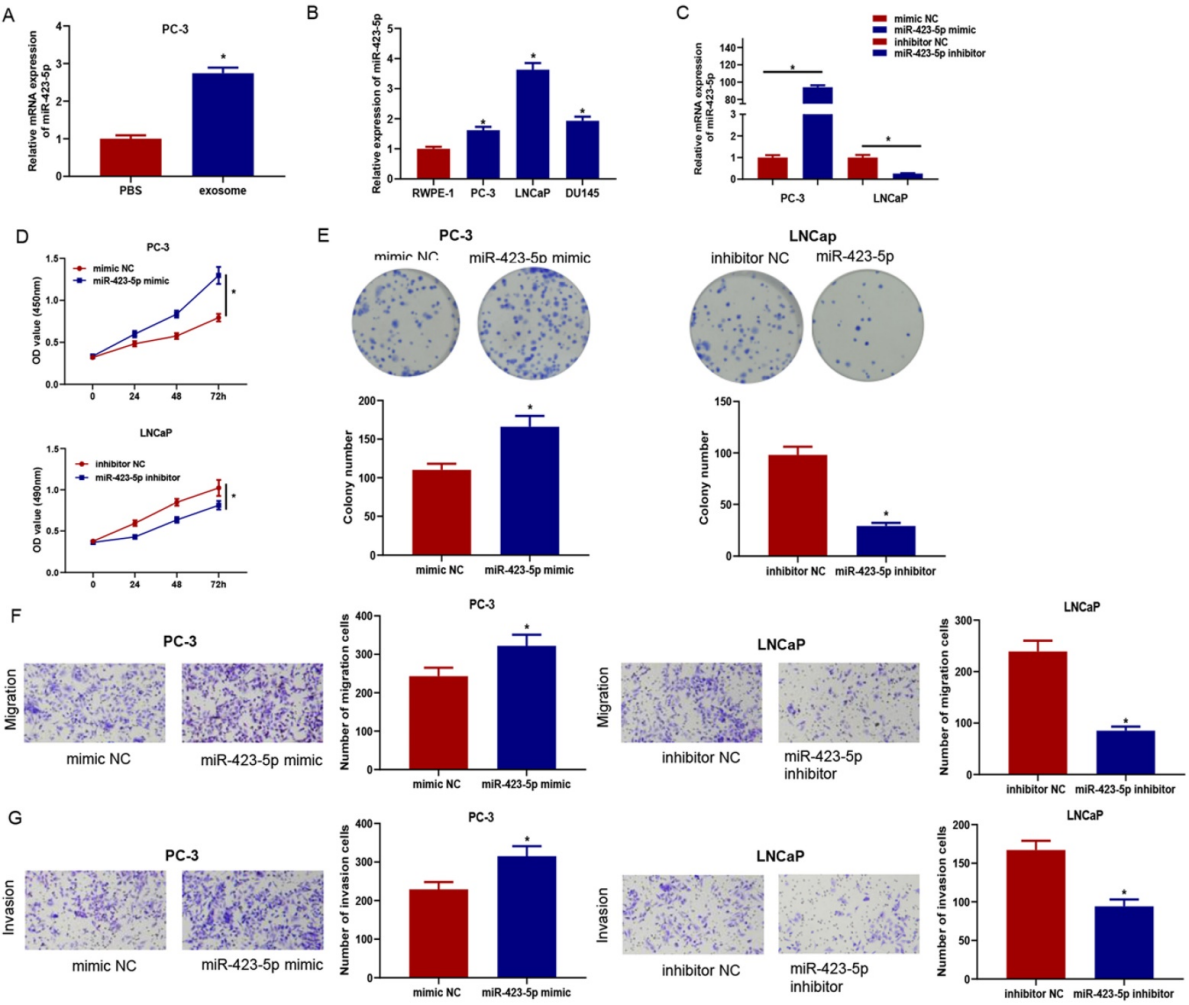
** MicroRNA-423-5p enhances proliferation, migration, and invasion of PCa.** MicroRNA-423-5p expression in A: PC-3 cells co-incubated with exosomes or PBS, B: PCa cell lines, C: Each treatment group. D: Cell viability in each group; E: Cell colony formation in each group; F: Migration in each group; G: Invasion in each group. (*p<0.05)

Previous experiments confirmed that exosomes could enter PC-3 cells and facilitate proliferation, migration, and invasion of cancer cells. qRT-PCR confirmed that the microRNA-423-5p level in PC-3 cells co-incubated with exosomes was remarkably higher than that in cells co-incubated with PBS (Figure 4A), indicating exosomal microRNA-423-5p might play vital roles in PCa progression. Therefore, qRT-PCR was employed to access microRNA-423-5p expression in PCa cells (PC-3, LNCaP, and DU145) (Figure 4B). PC-3 with microRNA-423-5p low expression and LNCaP with microRNA-423-5p high expression were selected as the objective cell lines. PC-3 cells were transfected with microRNA-423-5p mimic and mimic NC, while LNCaP cells were transfected with microRNA-423-5p inhibitor and inhibitor NC, respectively, followed by a series of cell function experiments. MicroRNA-423-5p expression in treatment groups was verified by qRT-PCR. The result showed that overexpressing microRNA-423-5p notably up-regulated microRNA-423-5p levels and silencing microRNA-423-5p remarkably down-regulated microRNA-423-5p levels (Figure 4C). CCK-8 and colony formation assays exhibited that the proliferative ability of PCa cells was enhanced by overexpression of microRNA-423-5p while this ability was reduced by the silence of microRNA-423-5p (Figure 4D-E). Transwell assay confirmed that migratory and invasive abilities were enhanced by overexpression of microRNA-423-5p while reduced by the silence of microRNA-423-5p (Figure 4F-G).

### MicroRNA-423-5p regulates the expression of FRMD3

**Figure 5:**
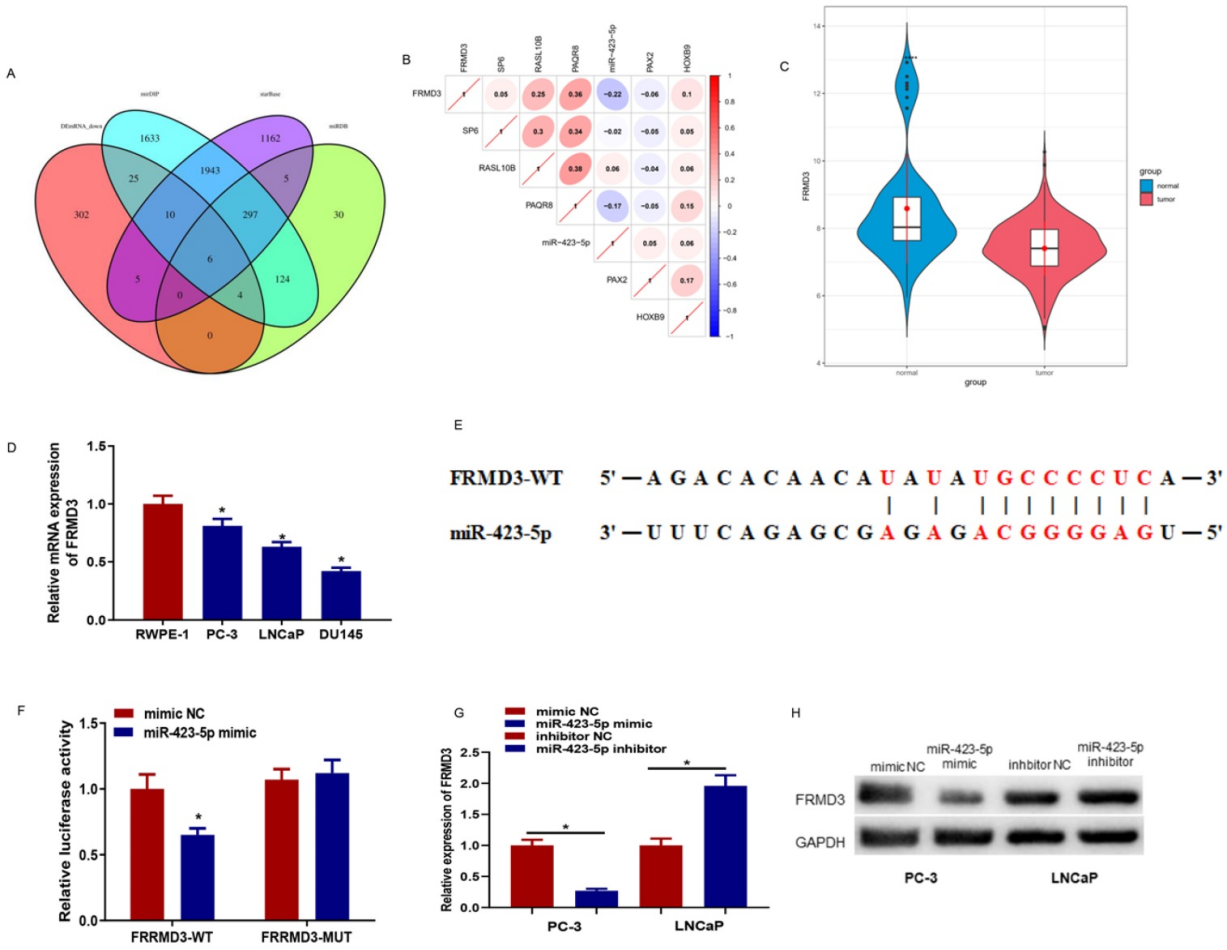
** MicroRNA-423-5p down-regulates FRMD3.** A: Venn diagram of the predicted downstream target mRNAs and down-regulated differential mRNAs of microRNA-423-5p; B: Pearson correlation analysis of FRMD3 and microRNA-423-5p; C: FRMD3 level in TCGA database. Green: normal samples, red: tumor samples; D: FRMD3 expression in each cell line; E: The predicted binding sites between microRNA-423-5p and FRMD3; F: Targeting relationship between microRNA-423-5p and FRMD3 was verified; G-H: FRMD3 mRNA and protein expression was tested.

The downstream targets of microRNA-423-5p were predicted using starBase, miRDB, and mirDIP databases. The prediction results were intersected with 352 down-regulated differential mRNAs, and 6 differential mRNAs possessing binding sites with microRNA-423-5p were obtained (Figure 5A). Pearson correlation analysis was performed, and its results indicated that FEMD3 was significantly negatively correlated with microRNA-423-5p with the highest correlation (Figure 5B). FEMD3 was significantly low expressed in PCa tissue (Figure 5C). qRT-PCR was applied to detect FEMD3 levels in PCa cell lines (PC-3, LNCaP, LNCaP, and DU145) and human epidermal cell lines (RWPE‐1). The result showed that FEMD3 was down-regulated in PCa cell lines (Figure 5D). We found that FEMD3 was an underlying target gene of microRNA-423-5p via bioinformatics prediction, the two genes had binding sequences (Figure 5E). The binding relationship was validated by dual-luciferase assay, and the results showed that microRNA-423-5p mimic significantly reduced the viability of FRMD3-WT 3'-UTR, while the viability of FRMD3-MUT 3'-UTR remained the same (Figure 5F). To identify the regulatory role of microRNA-423-5p on FRMD3, FRMD3 expression was evaluated by qRT-PCR and Western Blot. Compared with corresponding control groups, FRMD3 mRNA and protein levels in the microRNA-423-5p mimic group were notably reduced and their expression in the microRNA-423-5p inhibitor group was significantly elevated (Figure 5G-H). These results demonstrated that FRMD3 was the target of microRNA-423-5p, and it was negatively modulated by microRNA-423-5p at the expression level.

### MicroRNA-423-5p targeting FRMD3 modulates proliferation, migration, and invasion of PCa cells

**Figure 6:**
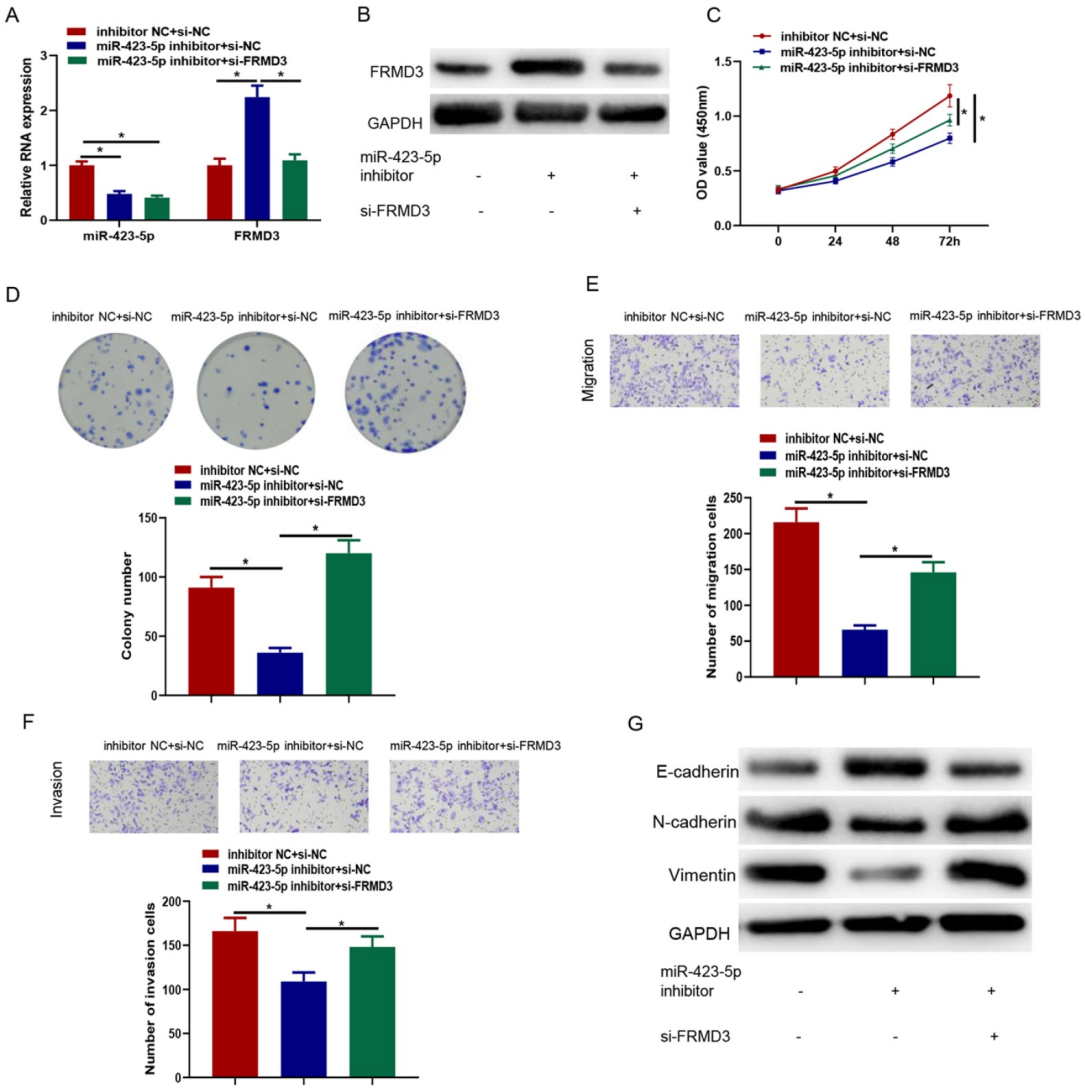
** MicroRNA-423-5p targeting FRMD3 modulates proliferation, migration, and invasion of PCa cells.** A: MicroRNA-423-5p and FRMD3 expression; B: FRMD3 protein expression; C: Cell viability; D: Colony formation; E-F: Cell migration and invasion; G: EMT-related protein expression. (*p<0.05)

It was crucial to identify whether FRMD3 affected the effects of microRNA-423-5p on promoting PCa. Inhibitor NC+si-NC, microRNA-423-5p inhibitor+si-NC, and microRNA-423-5p inhibitor+si-FRMD3 were co-transfected in LNCaP cells, respectively, followed by cell function assays. The levels of microRNA-423-5p and FRMD3 in each transfected group were measured by qRT-PCR. Compared with negative control, inhibiting microRNA-423-5p significantly reduced microRNA-423-5p expression; single repressing of microRNA-423-5p up-regulated FRMD3 expression. Simultaneously suppressing microRNA-423-5p and FRMD3 recovered the expression of FRMD3 but it was still downregulated in comparison with the single repressing microRNA-423-5p group (Figure 6A). Western blot confirmed that FRMD3 protein expression was reduced by co-transfected microRNA-423-5p inhibitor and si-FRMD3 compared with single transfection of microRNA-423-5p inhibitor (Figure 6B). CCK-8 and colony formation assays exhibited that silencing FRMD3 expression also partially reversed the blocked effect of the microRNA-423-5p inhibitor on cell growth and proliferation (Figure 6C-D). Transwell assay showed that silencing FRMD3 expression partially counteracted the suppressive effect of the microRNA-423-5p inhibitor on cell migration and invasion (Figure 6E-F). Moreover, we found that microRNA-423-5p inhibitor decreased protein expression of Vimentin and N-cadherin, and increased E-cadherin protein expression, while the silence of FRMD3 could reverse such effects (Figure 6G). These data indicated that microRNA-423-5p targeted FRMD3 to regulate the progression of PCa cells.

### Exosomes and microRNA-423-5p enhance PCa growth *in vivo*

**Figure 7:**
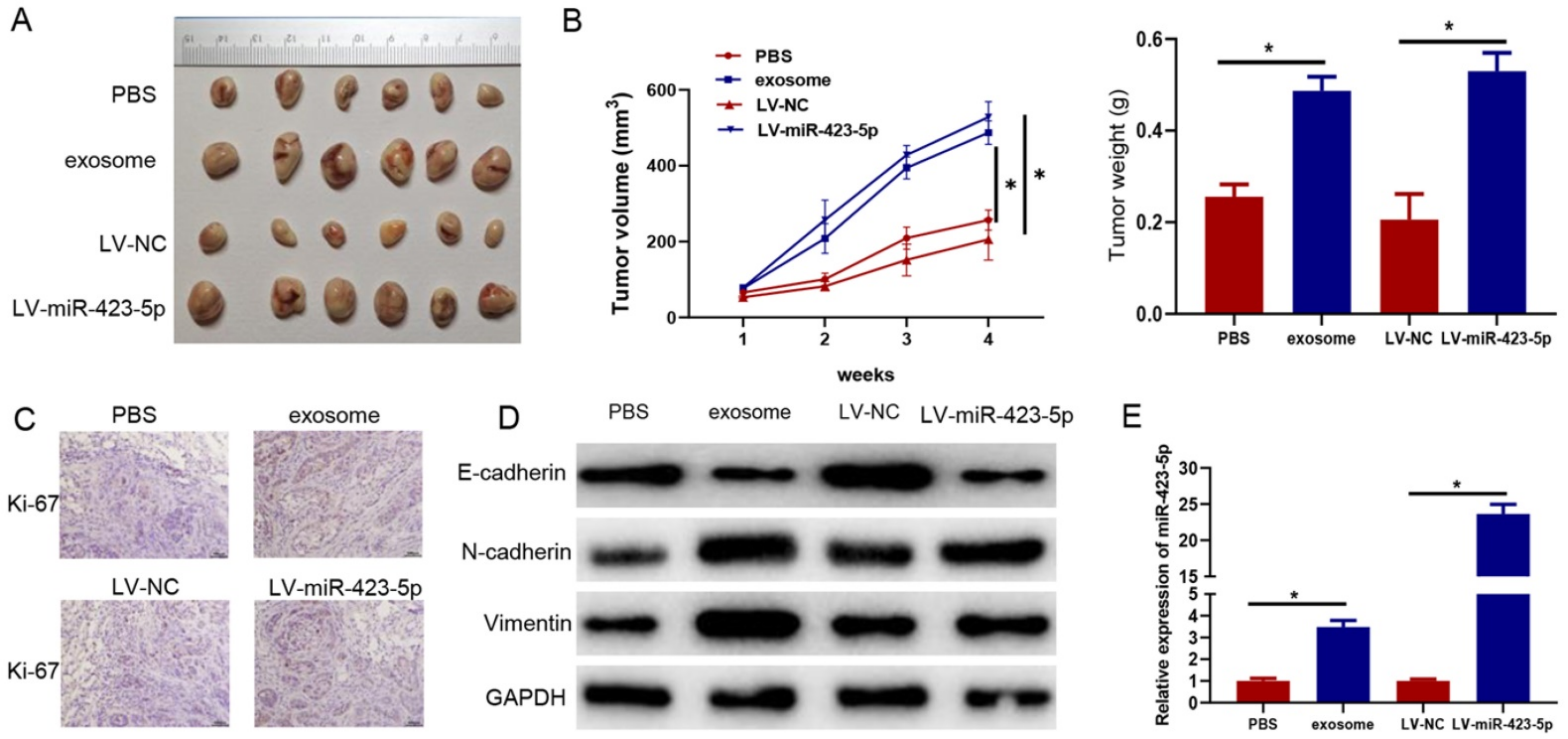
** Blood exosomes bearing microRNA-423-5p promote PCa growth *in vivo*.** A: Volume of subcutaneous tumor in each group; B: The appearance and weight of subcutaneous tumors in each group; C: Ki-67 expression (bars=50 µM); D: Expression of EMT-related proteins; E: MicroRNA-423-5p expression in tumor tissues in each group. (* *p*<0.05)

Subsequently, PC-3 cells were pretreated with blood exosomes carrying microRNA-423-5p/PBS and LV-miR-423-5p mimic/LV-NC. The impacts of exosomes and microRNA-423-5p on the growth of subcutaneously implanted tumors were investigated in tumor xenograft mice models. The tumor volume and weight in the exosome group were higher than those in the PBS group, and they were dramatically higher in the LV-miR-423-5p mimic group than those in the LV-NC group (Figure 7A-B). In addition, immunohistochemical analysis showed that positive staining of Ki-67 was stronger in xenograft of the exosome group than that in the PBS group, and it was stronger in the LV-miR-423-5p mimic group compared with the LV-NC group (Figure 7C). Moreover, Western blot demonstrated that in tumor tissue, N-cadherin and Vimentin expression was prominently up-regulated, and E-cadherin expression was notably down-regulated in the exosome group compared with the PBS group. Overexpression of microRNA-423-5p could up-regulate N-cadherin and Vimentin expression in tumor tissue and down-regulate E-cadherin expression. These results suggested that blood exosomes carrying microRNA-423-5p promoted the EMT process of PCa cells (Figure 7D). qRT-PCR exhibited that microRNA-423-5p expression in tumor tissue in the exosome group was notably higher than that in the PBS group, and microRNA-423-5p expression in tumor tissue in the LV-miR-423-5p group was dramatically higher than that in LV-NC group (Figure 7E). Overall, blood exosomes and microRNA-423-5p enhanced PCa growth *in vivo*.

## Discussion

The fatality rate of PCa ranks high among male malignancies worldwide in the last few decades 25. Digital rectal examination and detection of changes in biological markers can diagnose PCa 26. However, most detective tools are not sensitive or specific enough, making it difficult to treat PCa effectively 27. Exosomes have obvious advantages as novel biomarkers as they can be easily separated from the body fluids of tumor patients 12,28. To find a more effective therapy for PCa, we attempted to illustrate the role of exosomal microRNA-423-5p in the malignant progression of PCa. It was found that microRNA-423-5p can affect EMT via down-regulating FRMD3, thus promoting cell progression of PCa.

First of all, bioinformatics analysis demonstrated that microRNA-423-5p was highly expressed in PCa peripheral blood samples and largely existed in blood. Thus, we speculated that microRNA-423-5p in the blood of PCa patients might originate from blood exosomes and could regulate the malignant progression of PCa. The results of exosome extraction and qRT-PCR, exosomal microRNA-423-5p expression was remarkably higher in PCa patients relative to that in healthy people, which were consistent with bioinformatics results. Huang Yang et al. 24 discovered that microRNA-423-5p is elevated in serum exosomes of patients with gastric cancer, and microRNA-423-5p level is notably associated with lymph node metastasis, indicating that microRNA-423-5p may act as an underlying biomarker for gastric cancer. MicroRNA-423-5p is up-regulated in gastric cancer and negatively modulates TFF1 expression, to participate in proliferation and invasion processes 29. MicroRNA-423 regulates the transition of G (1)/S and enhances cell growth via targeting p21Cip1/Waf1 30. These results demonstrated that microRNA-423-5p functioned as an oncogene in cancer development. Therefore, to illustrate the role of microRNA-423-5p in the progression of PCa, microRNA-423-5p was overexpressed in PC-3 cells and was inhibited in LNCaP cells. Cell function assays and animal experiments exhibited that microRNA-423-5p affected EMT of PCa cells and promoted cell proliferation and motor ability.

Exosomes are important signaling devices mediating cellular communication 31. We observed that microRNA-423-5p was gathered in serum exosomes of PCa patients. It was explored that microRNA-423-5p-enriched exosomes were absorbed by PCa cells via exosome labeling and tracing experiments. Blood exosomes in PCa patients dramatically increase the proliferation, migration, and invasion of cancer cells, and ultimately promote their growth and motor ability, which is in line with the findings of Huang Yang et al. 24 The levels of EMT-related proteins implied that induction of EMT might be one of the reasons for enhanced invasiveness of PCa. FEMS3 was down-regulated in PCa and was the downstream target of microRNA-423-5p. Rescue experiments indicated that FRMD3 knockdown could weaken the repressive effect of the microRNA-423-5p inhibitor on the proliferation, migration, and invasion of cancer cells. In addition, it was reported that microRNA-423-5p enhances PCa cell proliferation and represses cell apoptosis via targeting GRIM-19 23. This study verified that microRNA-423-5p promoted PCa progression, indicating microRNA-423-5p promoted PCa development via targeting various genes.

Overall, microRNA-423-5p/FRMD3 enhanced cell proliferation, migration, and invasion in PCa. Exosomes could transport microRNA-423-5p to PCa cells and accelerate PCa growth and motor ability. A novel understanding of the role of exosomes in PCa was provided in this study, and it was expected to serve as a reference for PCa therapy. We will conduct more in-depth studies on microRNA-423-5p in serum exosomes in future studies. For instance, the follow-up of PCa patients was performed to explore the prognostic value.

## Supplementary Material

Supplementary table.

## Figures and Tables

**Table 1:** qRT-PCR primer sequences

Gene	Primer sequence (5'→ 3')
microRNA-423-5p	F: GCCTGAGGGGCAGAGAGC
	R: CCACGTGTCGTGGA GTC
FRMD3	F: TTTTCCCCAAGCAGTCACA
	R: TGCCCCCTGAGTTCATTTT
U6	F: CGCTTCGGCAGCACATATACTA
	R: CGCTTCACGAATTTGCGTGTCA
GAPDH	F: GCTCTCTGCTCCTCCTGTTC
	R: ACGACCAAATCCGTTGACTC

## References

[1] Li T, Sun X, Chen L (2020). Exosome circ_0044516 promotes prostate cancer cell proliferation and metastasis as a potential biomarker. J Cell Biochem.

[2] Center M M (2012). International variation in prostate cancer incidence and mortality rates. Eur Urol.

[3] Wang X (2019). Prostate carcinoma cell-derived exosomal MicroRNA-26a modulates the metastasis and tumor growth of prostate carcinoma. Biomed Pharmacother.

[4] Wang Y (2013). Should bone scan be performed in Chinese prostate cancer patients at the time of diagnosis?. Urol Int.

[5] Zhang J (2012). Trends in mortality from cancers of the breast, colon, prostate, esophagus, and stomach in East Asia: role of nutrition transition. Eur J Cancer Prev.

[6] Li Z (2016). et al. Exosomal microRNA-141 is upregulated in the serum of prostate cancer patients. Onco Targets Ther.

[7] Martínez-Reyes I, Chandel N S (2021). Cancer metabolism: looking forward. Nat Rev Cancer.

[8] Xie T (2019). Glioma stem cells reconstruct similar immunoinflammatory microenvironment in different transplant sites and induce malignant transformation of tumor microenvironment cells. J Cancer Res Clin Oncol.

[9] Shiao S L (2016). Regulation of prostate cancer progression by the tumor microenvironment. Cancer Lett.

[10] Colombo M (2014). Biogenesis, secretion, and intercellular interactions of exosomes and other extracellular vesicles. Annu Rev Cell Dev Biol.

[11] Gould S J, Raposo G (2013). As we wait: coping with an imperfect nomenclature for extracellular vesicles. J Extracell Vesicles.

[12] Li Y (2015). Circular RNA is enriched and stable in exosomes: a promising biomarker for cancer diagnosis. Cell Res.

[13] Melo S A (2014). Cancer exosomes perform cell-independent microRNA biogenesis and promote tumorigenesis. Cancer Cell.

[14] Janowska-Wieczorek A (2001). Platelet-derived microparticles bind to hematopoietic stem/progenitor cells and enhance their engraftment. Blood.

[15] Yu W (2021). Exosome-based liquid biopsies in cancer: opportunities and challenges. Ann Oncol.

[16] Bhagirath D (2018). microRNA-1246 Is an Exosomal Biomarker for Aggressive Prostate Cancer. Cancer Res.

[17] Li W (2020). Plasma exosomal miR-125a-5p and miR-141-5p as non-invasive biomarkers for prostate cancer. Neoplasma.

[18] Malla B (2018). Protocol for serum exosomal miRNAs analysis in prostate cancer patients treated with radiotherapy. J Transl Med.

[19] Du W (2018). LncRNA LINC00319 accelerates ovarian cancer progression through miR-423-5p/NACC1 pathway. Biochem Biophys Res Commun.

[20] Xu X (2022). Circ_FURIN knockdown assuages Testosterone-induced human ovarian granulosa-like tumor cell disorders by sponging miR-423-5p to reduce MTM1 expression in polycystic ovary syndrome. Reprod Biol Endocrinol.

[21] Sun X (2019). Long non-coding RNA LINC00968 reduces cell proliferation and migration and angiogenesis in breast cancer through up-regulation of PROX1 by reducing hsa-miR-423-5p. Cell Cycle.

[22] Wang X (2018). miR-423-5p Inhibits Osteosarcoma Proliferation and Invasion Through Directly Targeting STMN1. Cell Physiol Biochem.

[23] Lin H (2018). Inhibition of miR-423-5p suppressed prostate cancer through targeting GRIM-19. Gene.

[24] Yang H (2018). Exosomal miR-423-5p targets SUFU to promote cancer growth and metastasis and serves as a novel marker for gastric cancer. Mol Carcinog.

[25] Sung H (2021). Global cancer statistics 2020: GLOBOCAN estimates of incidence and mortality worldwide for 36 cancers in 185 countries. CA Cancer J Clin.

[26] Mottet N, van den Bergh R C N, Briers E (2021). EAU-EANM-ESTRO-ESUR-SIOG guidelines on prostate cancer—2020 update. Part 1: screening, diagnosis, and local treatment with curative intent. Eur Urol.

[27] Shao X (2017). Evaluation of expressed prostatic secretion and serum using surface-enhanced Raman spectroscopy for the noninvasive detection of prostate cancer, a preliminary study. Nanomedicine.

[28] Yu D (2022). Exosomes as a new frontier of cancer liquid biopsy. Mol Cancer.

[29] Liu J (2014). miRNA423-5p regulates cell proliferation and invasion by targeting trefoil factor 1 in gastric cancer cells. Cancer Lett.

[30] Lin J (2011). MicroRNA-423 promotes cell growth and regulates G(1)/S transition by targeting p21Cip1/Waf1 in hepatocellular carcinoma. Carcinogenesis.

[31] Denzer K (2000). Exosome: from internal vesicle of the multivesicular body to intercellular signaling device. J Cell Sci.

