# Supplementary material for: Blood circulating exosomes carrying microRNA-423-5p regulates cell progression in prostate cancer via targeting FRMD3
Source: J Cancer. 2022 Jul 18;13(9):2970–81. doi: 10.7150/jca.71706 (PMC9330460; doi:10.7150/jca.71706)
Supplement: Supplementary file 1 — Supplementary table. [file jcav13p2970s1.pdf]

Supplementary table 1 Clinic parameters of enrolled patients.

Sample	PCa Patients	Control group
No.	56	20
Median age (range, ys)	77.5 (56-85)	72.5 (60-83)
Diseases		
Pca	56	0
Urinary stones	0	11
UPJO	0	3
BPH	0	6
PCa risk groups$		
Low-risk	17	/
Intermediate-risk	24	/
High-risk	15	/

UPJO= ureteropelvic junction obstruction; BPH=benign prostatic hyperplasia; $ risk classifications according to EAU-EANM-ESTRO-ESUR-ISUP-SIOG-Guidelines-on-Prostate-Cancer-2021V3.
